# Association Between the Earliest CALLY Index at Diagnosis and Progression to End-Stage Kidney Disease in Patients with Microscopic Polyangiitis and Granulomatosis with Polyangiitis

**DOI:** 10.3390/medicina62071379

**Published:** 2026-07-17

**Authors:** Han Gyeol Lee, Jang Woo Ha, Oh Chan Kwon, Jihye Chung, Yong-Beom Park, Sang-Won Lee

**Affiliations:** 1 Department of Medicine, Yonsei University College of Medicine, Seoul 03722, Republic of Korea; 2 Division of Rheumatology, Department of Internal Medicine, Yonsei University College of Medicine, Yongin Severance Hospital, Yongin 16995, Gyeonggi-do, Republic of Korea; 3 Division of Rheumatology, Department of Internal Medicine, Yonsei University College of Medicine, Gangnam Severance Hospital, Seoul 06273, Republic of Korea; 4 Division of Rheumatology, Department of Internal Medicine, Yonsei University College of Medicine, 50-1 Yonsei-ro, Seodaemun-gu, Seoul 03722, Republic of Korea; 5 Institute for Immunology and Immunological Diseases, Yonsei University College of Medicine, Seoul 03722, Republic of Korea

**Keywords:** microscopic polyangiitis, granulomatosis with polyangiitis, CALLY index, end-stage kidney disease, renal outcome

## Abstract

*Background and Objectives*: This study aimed to determine whether the earliest C-reactive protein (CRP)-albumin-lymphocyte (CALLY) index at diagnosis was associated with progression to end-stage kidney disease (ESKD) during follow-up in patients with microscopic polyangiitis (MPA) and granulomatosis with polyangiitis (GPA). *Materials and Methods*: This study included 247 patients with MPA and GPA. The CALLY index was calculated as follows: serum albumin (g/L) × lymphocyte count (/mm^3^)/CRP (mg/L)/10,000. The earliest CALLY index was defined as the value calculated at diagnosis. ESKD, which occurred after diagnosis, was defined as a poor outcome in this study. *Results*: The median age of the 247 patients was 62.0 years and 64.4% were women. Among the 247 patients, 50 (20.2%) progressed to ESKD. The earliest CALLY index at diagnosis was significantly lower in patients with ESKD than those without ESKD (0.15 vs. 0.55). Receiver operating characteristic curve analysis showed that the earliest CALLY index had modest discriminatory performance for progression to ESKD, with an area under the curve of 0.663 and a cut-off value of 0.26. Patients with an earliest CALLY index ≤ 0.26 at diagnosis exhibited a significantly higher risk for progression to ESKD (relative risk 3.174) and a significantly reduced cumulative ESKD-free survival rate compared to those with an earliest CALLY index > 0.26 at diagnosis. *Conclusions*: This study has exploratory implications, as it is the first to report that the earliest CALLY index was associated with ESKD occurrence during follow-up in patients with MPA and GPA, despite its limited value as an independent predictor of ESKD.

## 1. Introduction

Antineutrophil cytoplasmic antibody (ANCA)-associated vasculitis (AAV) encompasses a group of vasculitides predominantly affecting small vessels, including capillaries, venules, and arterioles, although medium-sized arteries may also be involved. Histopathologically, AAV is typically associated with necrotizing inflammation and fibrinoid change in the vessel wall, with minimal or absent immune-complex deposition [1,2]. According to the integrated assessment of clinical manifestations, laboratory findings, imaging features, and histopathology, AAV is categorised into three major phenotypes: microscopic polyangiitis (MPA), granulomatosis with polyangiitis (GPA), and eosinophilic granulomatosis with polyangiitis (EGPA). MPA commonly affects the lungs and kidneys, leading to pulmonary fibrosis, interstitial lung disease, and glomerulonephritis. GPA commonly involves both the upper and lower respiratory systems, presenting with manifestations such as refractory otitis media, subglottic stenosis, and characteristic pulmonary abnormalities, including nodular lesions and cavitation, whereas EGPA frequently exhibits clinical manifestations of combined allergic and vasculitic features [3,4,5,6].

In terms of kidney involvement of AAV, renal manifestations can be observed in approximately 90–100% of MPA patients and approximately 50–80% of GPA patients. However, in patients with EGPA, renal manifestations may be observed less commonly than in those with the other two subtypes: they are higher in ANCA-positive patients than in ANCA-negative patients, but do not exceed 50% [7]. Progression to end-stage kidney disease (ESKD) has been reported in a relatively high proportion of patients with MPA and GPA within approximately 5 years of diagnosis [8,9,10]. Furthermore, initial myeloperoxidase (MPO)-ANCA positivity rather than proteinase 3 (PR3)-ANCA, sex, age, serum creatinine and/or its rapid rise, and renal histological findings at diagnosis have been considered significant risk factors for ESKD [10,11]. Given the significant number of patients who progress to ESKD, the small proportion of patients who discontinue renal replacement therapy after ESKD occurrence, and the increased risk of systemic complications of ESKD itself, developing indices for predicting progression to ESKD at diagnosis may be clinically important in real clinical settings [8,11].

The C-reactive protein (CRP)-albumin-lymphocyte (CALLY) index, which consists of CRP, serum albumin, and lymphocyte count, has been proven to predict poor outcomes in several malignant diseases [12,13]. Beyond malignant disorders, the potential relevance of the CALLY index to renal disease has also been suggested. Although its direct association with kidney function has not yet been evaluated, a recent population-based study found that lower CALLY index values were associated with a higher risk of cardiorenal syndrome, indirectly supporting a possible link between the CALLY index and renal functional impairment [14]. Because the individual components of the CALLY index may reflect both the severity of glomerulonephritis and the overall disease activity of MPA and GPA, the baseline CALLY index obtained at diagnosis may be associated with subsequent renal outcomes in these patients. However, to date, no study has addressed this issue. This study evaluated the prognostic relevance of the earliest CALLY index for ESKD development during follow-up among patients diagnosed with MPA or GPA.

## 2. Materials and Methods

### 2.1. Study Patients

We selected 247 patients with MPA and GPA from the Severance Hospital ANCA-associated VasculitidEs (SHAVE) cohort, a single-centre observational cohort of Korean patients with AAV, and included them in the present study. All patients met the inclusion criteria as follows: (i) first diagnosis of MPA and GPA was made by specialised rheumatologists at this tertiary University Hospital from November 2005 to March 2024; (ii) fulfilment of the 2007 European Medicine Agency algorithm for AAV, and the 2012 revised international Chapel Hill Consensus Conference nomenclature of vasculitides at diagnosis [1,2]; (iii) fulfilment of the 2022 ACR/European Alliance of Associations for Rheumatology (EULAR) classification criteria for AAV at diagnosis [4,5,6]; (iv) availability of sufficiently comprehensive medical records to obtain clinical information from the time of diagnosis through the most recent follow-up visit; (v) availability of the results of ANCA tests performed within four weeks before and two weeks after MPA and GPA diagnosis [15]; (vi) follow-up period for at least 6 months; (vii) absence of major concurrent comorbidities, including malignancy diagnosed within 3 years of MPA or GPA diagnosis and active infection at the time of diagnosis [15,16]; and (viii) no exposure to immunosuppressive drugs within 4 weeks before MPA and GPA diagnosis.

### 2.2. Ethical Statement

The study protocol received approval from the Severance Hospital Institutional Review Board (IRB No. 4-2016-0901), and all study procedures complied with the principles of the Declaration of Helsinki. Each participant provided written informed consent upon entry into the SHAVE cohort and before collection of blood samples. The IRB waived the need for written informed consent if it was previously obtained while entering the SHAVE cohort.

### 2.3. Clinical Data at AAV Diagnosis

Demographic data included age, sex, body mass index (BMI), and smoking history. Regarding AAV-related variables, AAV subtype, ANCA type and positivity, and AAV-specific indices were also assessed. Serum MPO-ANCA and PR3-ANCA levels were determined by antigen-specific immunoassays, while perinuclear (P)-ANCA and cytoplasmic (C)-ANCA patterns were assessed using indirect immunofluorescence [17]. Based on the 2022 ACR/EULAR classification criteria for MPA, and GPA [4,5,6,15], MPO-ANCA, PR3-ANCA, P-ANCA, and C-ANCA were recognised as the results of ANCA tests [18]. The Birmingham Vasculitis Activity Score (BVAS) and the Five-Factor Score (FFS) were obtained as AAV-specific indices [19,20]. Among the laboratory results, erythrocyte sedimentation rate (ESR) and CRP levels were recorded as acute-phase reactants [21]. Comorbidities before AAV diagnosis, including hypertension, type 2 diabetes mellitus, and dyslipidaemia were also reviewed [22].

### 2.4. CALLY Index

The CALLY index was calculated using the following equation: serum albumin (g/L) × lymphocyte count (/mm^3^)/CRP (mg/L)/10,000 [12,13]. The earliest CALLY index was defined as the value of the CALLY index calculated using the laboratory results at diagnosis.

### 2.5. Clinical Data During the Follow-Up Period After MPA and GPA Diagnosis

In this study, progression to ESKD was considered an adverse renal outcome of MPA and GPA and was defined as the need for renal replacement therapy due to chronic kidney disease [23]. For patients who developed ESKD, the observation period was calculated from the date of MPA or GPA diagnosis to the initiation of renal replacement therapy. For those who did not develop ESKD, follow-up extended from diagnosis to the most recent clinical visit. We also collected information on the use of glucocorticoids and immunosuppressive agents for the treatment of MPA or GPA during follow-up.

### 2.6. Statistical Analyses

Statistical analyses were conducted using IBM SPSS Statistics for Windows, version 26 (IBM Corp., Armonk, NY, USA). Continuous variables are presented as medians with interquartile ranges, and categorical variables as counts and percentages. Differences in continuous variables between two groups were evaluated using the Mann–Whitney U test. Receiver operating characteristic (ROC) curve analysis was performed to assess discriminatory ability, with the area under the curve (AUC) calculated accordingly. The optimal cut-off value was defined as the point yielding the highest combined sensitivity and specificity. The relative risk (RR) of progression to ESKD according to this cut-off was estimated using a contingency table and assessed with the chi-square test. ESKD-free survival was compared between groups using Kaplan–Meier analysis and the log-rank test. The proportional hazards assumption was assessed graphically using log-minus-log survival plots, and no marked violation was observed. Given the limited number of ESKD events, the multivariable Cox proportional hazards model was restricted to four clinically relevant covariates selected a priori: the earliest CALLY index, baseline serum creatinine, BVAS, and MPO-ANCA positivity to estimate hazard ratios (HRs) for ESKD during follow-up. Multicollinearity among the covariates included in the final multivariable Cox model was assessed using tolerance values and variance inflation factors. A two-sided *p* value < 0.05 was considered statistically significant.

## 3. Results

### 3.1. Baseline and Follow-Up Characteristics According to Progression to ESKD

The median age of the 247 patients was 62.0 years, with 64.4% being women. The median BMI was 22.5 kg/m2, and six patients were ex-smokers. Among the 247 patients, 178 (72.1%) and 69 (27.9%) were diagnosed with MPA and GPA, respectively. MPO-ANCA (or P-ANCA) and PR3-ANCA (or C-ANCA) were detected in 189 (76.5%) and 41 (16.6%) patients, respectively. The median BVAS, FFS, ESR, and CRP levels were 12.0, 1.0, 62.0 mm/h, and 13.2 mg/L, respectively. In addition, hypertension, type 2 diabetes mellitus, and dyslipidaemia were present in 108, 66, and 43 patients, respectively. The median earliest CALLY index, calculated at diagnosis, was 0.43. Of the 247 patients, 50 (20.2%) progressed to ESKD over a median follow-up duration of 39.0 months. Regarding medications administered for MPA and GPA treatment, glucocorticoids were prescribed to 231 patients. The most frequently administered immunosuppressive drug was cyclophosphamide (51.4%), followed by azathioprine (51.0%), mycophenolate mofetil (27.5%), and rituximab (21.5%). Compared with patients who did not progress to ESKD, those who progressed to ESKD had a lower BMI, higher BVAS and FFS, lower haemoglobin and platelet counts, higher blood urea nitrogen and serum creatinine levels, lower serum total protein, serum albumin, and eGFR, and more frequent proteinuria and hematuria at diagnosis. In addition, CRP levels and the frequency of hypertension were higher in patients who progressed to ESKD. The earliest CALLY index at diagnosis was significantly lower in patients with ESKD progression than in those without ESKD progression (Table 1).

### 3.2. Comparison of the Earliest CALLY Index

**Figure 1:**
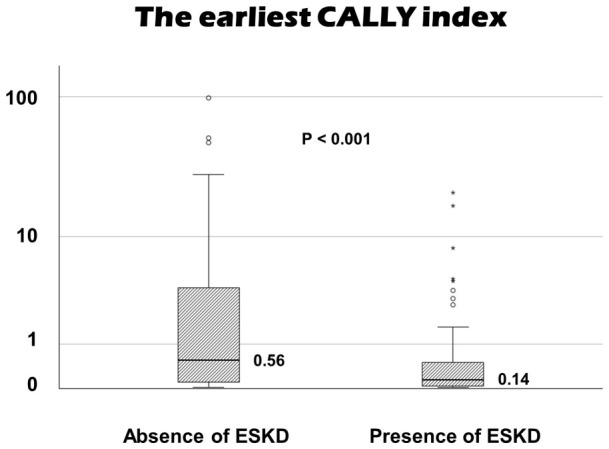
Comparison of the earliest CALLY index according to progression to ESKD. Boxplots show the distribution of the earliest CALLY index in patients with and without progression to ESKD. Boxes indicate interquartile ranges, horizontal lines indicate medians, and whiskers indicate 1.5 times the interquartile range. Circles indicate outliers (1.5–3 × interquartile range [IQR] from the box), and asterisks indicate extreme outliers (>3 × IQR). CALLY: C-reactive protein-albumin-lymphocyte; ESKD: end-stage kidney disease.

The earliest CALLY index at diagnosis was significantly lower in patients who progressed to ESKD than in those who did not progress to ESKD (0.15 [0.03–0.54] vs. 0.55 [0.10–4.05], *p* < 0.001) (Figure 1). Because the CALLY index showed a skewed distribution, the data are presented as medians with interquartile ranges and illustrated using a boxplot.

These findings indicate that a lower earliest CALLY index at diagnosis was associated with subsequent progression to ESKD in patients with MPA and GPA.

### 3.3. Relative Risk for ESKD and Cumulative ESKD-Free Survival Rates

**Figure 2:**
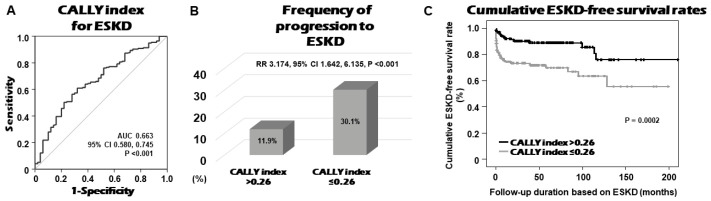
Assessment of relative risk for ESKD and comparison of cumulative ESKD-free survival rates. (**A**) A ROC curve of the CALLY index for progression to ESKD, (**B**) relative risk for progression to ESKD according to an earliest CALLY index ≤ 0.26, and (**C**) cumulative ESKD-free survival rates according to an earliest CALLY index ≤ 0.26. ESKD: end-stage kidney disease; ROC: receiver operating characteristic; AUC: area under the curve; CALLY: C-reactive protein-albumin-lymphocyte.

ROC curve analysis showed that the earliest CALLY index at diagnosis had modest discriminatory performance for ESKD progression, with an AUC of 0.663 (95% confidence interval [CI] 0.580, 0.745). The internally derived cut-off value for progression to ESKD was determined as 0.26 (with sensitivity and specificity of 60.4% and 68.0%, respectively) (Figure 2A).

When patients were divided into two groups according to the cut-off value of the earliest CALLY index calculated at diagnosis of 0.26, during the follow-up period, progression to ESKD was observed in patients with the earliest CALLY index ≤ 0.26 more frequently than in those with an earliest CALLY index > 0.26 (30.1% vs. 11.9%, *p* < 0.001). Additionally, patients with an earliest CALLY index ≤ 0.26 exhibited a significantly higher risk for progression to ESKD compared to those without (RR 3.174, 95% CI 1.642, 6.135, *p* < 0.001) (Figure 2B).

### 3.4. Comparison of Cumulative ESKD-Free Survival Rates

Patients with an earliest CALLY index ≤ 0.26 demonstrated a significantly lower cumulative ESKD-free survival rate than those with an earliest CALLY index > 0.26 (Figure 2C). These survival analyses further support an association between a lower earliest CALLY index and subsequent progression to ESKD.

### 3.5. Discriminatory Performance of the Inverse CALLY Index and Baseline Serum Creatinine

**Figure 3:**
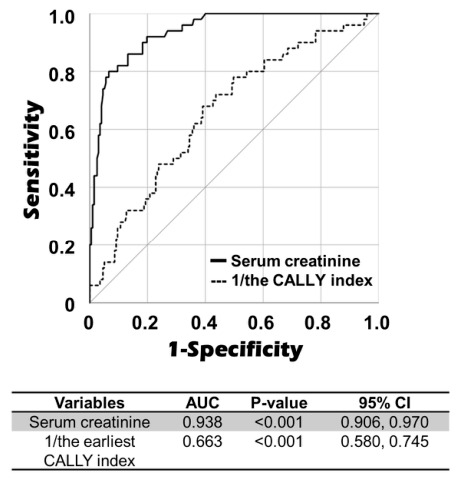
Comparison of the areas under the receiver operating characteristic curves for progression to ESKD. Baseline serum creatinine showed substantially higher discriminatory performance for ESKD progression than the inverse form of the earliest CALLY index. The inverse CALLY index was used because lower values of the earliest CALLY index were associated with a higher frequency of ESKD progression. AUC: area under the curve; CI: confidence interval; CALLY: C-reactive protein-albumin-lymphocyte; ESKD: end-stage kidney disease.

We further compared the discriminatory performance of baseline serum creatinine and the inverse form of the earliest CALLY index for progression to ESKD during follow-up. Baseline serum creatinine showed a higher AUC for ESKD progression than the inverse CALLY index (AUC 0.938, 95% CI 0.906, 0.970 vs. AUC 0.663, 95% CI 0.580, 0.745, respectively) (Figure 3). The absolute difference in AUC between baseline serum creatinine and the inverse CALLY index was 0.275. These findings indicate that although the earliest CALLY index was associated with ESKD progression, its discriminatory performance was substantially lower than that of baseline serum creatinine.

### 3.6. Univariable Cox Proportional Hazard Analyses for Progression to ESKD

In univariable Cox analysis, initial BMI (HR 0.863), MPO-ANCA (or P-ANCA) (HR 2.462), BVAS (HR 1.086), FFS (HR 1.975), haemoglobin (HR 0.653), blood urea nitrogen (HR 1.029), serum creatinine (HR 1.523), serum albumin (HR 0.518), CRP (HR 1.006), and hypertension (HR 2.274) at diagnosis exhibited the significant association with progression to ESKD during the follow-up period among patients with MPA and GPA. Additionally, in univariable Cox analysis, the earliest CALLY index was significantly associated with progression to ESKD during follow-up in patients with MPA and GPA, with the HR calculated per one-unit increase in the CALLY index (HR 0.902, 95% CI 0.816–0.997, *p* = 0.043) (Table 2).

### 3.7. Multivariable Cox Proportional Hazard Analyses for Progression to ESKD

Multivariable Cox regression analysis was performed to evaluate whether the earliest CALLY index at diagnosis was independently associated with progression to ESKD during follow-up. Given the limited number of ESKD events, the multivariable model was restricted to four clinically relevant covariates selected a priori: the earliest CALLY index, baseline serum creatinine, BVAS, and MPO-ANCA positivity at diagnosis. No relevant multicollinearity was observed among the covariates included in the final multivariable Cox model. In multivariable Cox analysis, baseline serum creatinine at diagnosis was independently associated with progression to ESKD during follow-up in patients with MPA and GPA (HR 1.541, 95% CI 1.410, 1.683, *p* < 0.001). BVAS at diagnosis was also independently associated with progression to ESKD (HR 1.069, 95% CI 1.028, 1.111, *p* < 0.001). However, the earliest CALLY index at diagnosis was not independently associated with progression to ESKD after adjustment for baseline serum creatinine, BVAS, and MPO-ANCA positivity (Table 2).

## 4. Discussion

Given the potential of the parameters constituting the CALLY index to reflect kidney inflammation, this study investigated whether the earliest CALLY index calculated at diagnosis was associated with subsequent progression to ESKD during follow-up in patients with MPA and GPA. The key findings are as follows: first, the earliest CALLY index at diagnosis was significantly lower in patients with progression to ESKD than those without ESKD during the follow-up period; second, ROC curve analysis showed a modest discriminatory performance of the earliest CALLY index at diagnosis for progression to ESKD and determined an internally derived cut-off of an earliest CALLY index as 0.26; third, patients with an earliest CALLY index ≤ 0.26 at diagnosis exhibited a significantly higher risk for progression to ESKD (RR 3.174) and a significantly reduced cumulative ESKD-free survival rate than those with an initial CALLY index > 0.26; however, fourth, the earliest CALLY index at diagnosis was not independently associated with progression to ESKD in patients with MPA and GPA. Therefore, the present findings should be interpreted as exploratory and hypothesis-generating, suggesting that the earliest CALLY index may be a complementary laboratory marker associated with renal outcome.

The present study explored potential explanations for the observed association between the earliest CALLY index at diagnosis and subsequent progression to ESKD in patients with MPA and GPA. The first inference is that there might be an association between each parameter composing the CALLY index and active renal vasculitis of a potential risk of ESKD occurrence: (i) in terms of serum albumin, a reduced level of serum albumin may occur directly due to loss of serum albumin by proteinuria, and indirectly as an acute-phase reactant reflecting the activity of vasculitis, particularly glomerulonephritis [10,21,24,25]; (ii) in terms of lymphocyte count, a decreased number of lymphocytes has been reported to be related to acute glomerulonephritis [26]; and (iii) in terms of CRP levels, an elevated level of CRP was also known associated with the activity of ANCA-associated renal vasculitis [27]. The earliest CALLY index was correlated with serum creatinine at diagnosis, suggesting that a lower CALLY index may partly reflect worse baseline renal function. The weak but statistically significant inverse correlation between the earliest CALLY index and serum creatinine at diagnosis (r = −0.148, *p* = 0.020) suggests that a lower CALLY index may partly reflect worse baseline renal function. Therefore, the association between the earliest CALLY index and ESKD progression may be partly explained by its relationship with baseline inflammation, nutritional status, lymphocyte-related immune alterations, and renal function. However, the present data do not establish that the CALLY index directly reflects the severity of renal vasculitis.

Despite these mechanistic advantages, in multivariable Cox analysis for progression to ESKD, the earliest CALLY index calculated at diagnosis was not found to have a significant independent association with progression to ESKD, whereas serum creatinine and BVAS at diagnosis showed a significant independent association with ESKD (Table 2). Additionally, the ROC curve analysis comparing progression to ESKD during follow-up revealed that the AUC of the inverse form of the earliest CALLY index at diagnosis (1 divided by the CALLY index) (area 0.663, 95% CI 0.580, 0.745) did not surpass that of serum creatinine at diagnosis (area 0.938, 95% CI 0.906, 0.970) (Figure 3). Nevertheless, the association between the earliest CALLY index at diagnosis and subsequent progression to ESKD may be partly explained by several factors. First, the CALLY index was associated with ESKD occurrence, which can buffer the impact of drastic external factors, as it is composed of three parameters reflecting active vasculitis multi-dimensionally [12,13]. Second, the CALLY index paradoxically does not include serum creatinine levels as its parameter. This has the advantage of being able to flexibly address the reversibility of temporary renal dysfunction due to various causes [28]. Therefore, although the earliest CALLY index at diagnosis showed lower discriminatory performance for progression to ESKD than serum creatinine at diagnosis, it may still have exploratory relevance as a complementary marker.

We also investigated whether the earliest CALLY index calculated at diagnosis was associated with non-renal outcomes, including cerebrovascular accident (CVA) and acute coronary syndrome (ACS), during follow-up in patients with MPA and GPA. Among the 247 patients, 17 and eight experienced CVA and ACS, respectively, after diagnosis. However, neither CVA nor ACS showed significant differences in cumulative survival rates according to the cut-off of the earliest CALLY index for progression to ESKD (Figure S1). Taken together, these exploratory findings suggest that the earliest CALLY index calculated at diagnosis may be more closely related to subsequent ESKD progression than to CVA or ACS during follow-up in patients with MPA and GPA.

In this study, the earliest CALLY index at diagnosis was significantly and inversely correlated with cross-sectional BVAS in patients with MPA and GPA (r = −0.193, *p* = 0.002). We also examined the difference in the earliest CALLY index according to the nine systemic items of BVAS, which are categorised based on the major organs affected by MPA and GPA. The most frequently affected major organ was the kidneys (67.2%), followed by the lungs (62.3%). Of the nine items, the median values of the earliest CALLY index in patients with general (0.14 vs. 0.98, *p* < 0.001), pulmonary (0.30 vs. 0.47, *p* = 0.024), cardiovascular (0.16 vs. 0.53, *p* = 0.020), and renal (0.24 vs. 1.83, *p* < 0.001) manifestations were significantly lower than those without (Table S1). The relationship between the presence of general and renal manifestations and the decreased values of the earliest CALLY index was expected based on the results of this study; however, the inverse correlation of the earliest CALLY index with clinical manifestations due to cardiovascular and pulmonary involvement may have clinical implications.

The strength of this study is that it is the first to demonstrate that the earliest CALLY index developed using the results of routinely performed laboratory tests has potential relevance to ESKD progression during the follow-up period in patients with MPA and GPA. This study has several limitations. Most importantly, the earliest CALLY index was not independently associated with ESKD after adjustment for baseline creatinine and BVAS, and its discriminatory performance was modest. Therefore, the present findings should not be interpreted as evidence that the CALLY index is an established prognostic tool. In addition, we did not formally evaluate whether the earliest CALLY index improved predictive performance beyond established renal prognostic markers using changes in the concordance statistic, net reclassification improvement, or integrated discrimination improvement. Thus, the incremental value of the CALLY index beyond conventional renal-severity variables remains uncertain. In addition, the cut-off value of ≤0.26 was derived from and applied to the same cohort. Therefore, the cut-off-based estimates, including ESKD frequency, relative risk, and ESKD-free survival, may be affected by optimism bias. This threshold requires external validation before it can be considered for clinical use. Another important limitation is the limited number of ESKD events. Because only 50 patients progressed to ESKD, the multivariable Cox regression model may still be vulnerable to overfitting, although we reduced the number of covariates to four clinically relevant variables selected a priori. Therefore, the adjusted estimates should be interpreted as exploratory rather than confirmatory. Third, this study was conducted retrospectively at a single centre, and incomplete clinical data restricted further subgroup and sensitivity analyses. In particular, complete kidney histopathological classification was not available for all patients; therefore, the present study could not fully adjust for biopsy-based renal prognostic categories. Although the inclusion of Korean patients from a single tertiary centre may have reduced ethnic and geographic heterogeneity, this design may limit the generalisability of the findings. Finally, this study evaluated only the earliest CALLY index at diagnosis and did not assess serial changes in the CALLY index or their parallel relationships with renal outcomes and disease activity during follow-up. Future prospective studies with larger cohorts, more ESKD events, complete renal histopathological data, treatment information, and serial CALLY index measurements are needed to validate the clinical relevance of the CALLY index as a complementary laboratory marker associated with ESKD progression in patients with MPA and GPA.

## 5. Conclusions

This study has potential clinical relevance in that it is the first to show that the earliest CALLY index, calculated from routinely available laboratory tests, was associated with ESKD occurrence during follow-up in patients with MPA and GPA, although it was not independently associated with ESKD after adjustment for baseline creatinine and BVAS.

## Figures and Tables

**Table 1:** Baseline characteristics of patients with MPA and GPA according to progression to ESKD. (*N* = 247).

Variables	Total (*N* = 247)	Non-ESKD (*N* = 197)	ESKD (*N* = 50)	*p*-Value
***At diagnosis***				
**Demographic data**				
Age (years)	62.0 (51.0–71.0)	62.0 (51.0–70.0)	62.0 (54.0–73.0)	0.283
Male sex (*N*, (%))	88 (35.6)	74 (37.6)	14 (28.0)	0.207
Female sex (*N*, (%))	159 (64.4)	123 (62.4)	36 (72.0)	
BMI (kg/m^2^)	22.5 (20.3–24.8)	22.7 (20.8–25.1)	21.1 (19.4–23.1)	<0.001
Ex-smoker (*N*, (%))	6 (2.4)	5 (2.5)	1 (2.0)	>0.999
**AAV subtype (** ***N*** **, (%))**				
MPA	178 (72.1)	140 (71.1)	38 (76.0)	0.487
GPA	69 (27.9))	57 (28.9)	12 (24.0)	
**ANCA type and positivity (** ***N*** **, (%))**				
MPO-ANCA (or P-ANCA) positivity	189 (76.5)	146 (74.1)	43 (86.0)	0.077
PR3-ANCA (or C-ANCA) positivity	41 (16.6)	35 (17.8)	6 (12.0)	0.328
**AAV-specific indices**				
BVAS	12.0 (6.0–18.0)	11.0 (6.0–16.0)	17.0 (10.0–22.0)	<0.001
FFS	1.0 (1.0–2.0)	1.0 (0–2.0)	2.0 (1.0–3.0)	<0.001
**Laboratory results**				
White blood cell count (/mm^3^)	8840.0 (6350.0–12,330.0)	8775.0 (6287.5–12,285.0)	9040.0 (6420.0–13,430.0)	0.454
Haemoglobin (g/dL)	11.1 (9.5–12.9)	11.7 (10.0–13.1)	9.3 (8.0–10.8)	<0.001
Platelet count (×1000/mm^3^)	294.0 (226.0–388.0)	308.0 (230.5–394.3)	255.0 (181.0–324.0)	0.006
Blood urea nitrogen (mg/dL)	18.6 (14.0–35.8)	16.8 (12.8–23.1)	42.6 (33.5–58.8)	<0.001
Serum creatinine (mg/dL)	0.9 (0.7–2.0)	0.8 (0.6–1.2)	4.3 (2.7–6.2)	<0.001
Serum total protein (g/dL)	6.8 (6.2–7.3)	6.8 (6.3–7.3)	6.3 (5.7–6.9)	<0.001
Serum albumin (g/dL)	3.7 (3.1–4.3)	3.9 (3.2–4.3)	3.3 (2.9–3.8)	<0.001
eGFR (mL/min/1.73 m^2^)	78.8 (29.7–100.8)	93.5 (60.9–104.6)	11.8 (8.2–21.2)	<0.001
Proteinuria (*N*, (%))	106 (42.9)	69 (35.2)	37 (75.5)	<0.001
Hematuria (*N*, (%))	138 (55.9)	98 (50.0)	40 (81.6)	<0.001
**Acute phase reactants**				
ESR (mm/hr)	62.0 (24.5–101.0)	58.5 (23.0–103.0)	69.0 (40.0–96.0)	0.592
CRP (mg/L)	13.2 (1.7–60.3)	9.3 (1.4–50.8)	20.9 (6.1–84.7)	0.004
**Comorbidities (** ***N*** **, (%))**				
Hypertension	108 (43.7)	77 (39.1)	31 (62.0)	0.004
Type 2 diabetes mellitus	66 (26.7)	53 (26.9)	13 (26.0)	0.897
Dyslipidaemia	43 (17.4)	33 (16.8)	10 (20.0)	0.588
**CALLY index**	0.43 (0.06–3.60)	0.55 (0.10–4.05)	0.15 (0.03–0.54)	<0.001
***During the follow-up period***				
**ESKD (** ***N*** **, (%))**				
ESKD	50 (20.2)	0 (0)	50 (100.0)	
Follow-up duration based on ESKD (month)	39.0 (8.8–77.3)	51.1 (18.1–83.3)	3.7 (0.4–17.3)	<0.001
**Medications (** ***N*** **, (%))**				
Glucocorticoids	231 (93.5)	182 (92.4)	49 (98.0)	0.206
Cyclophosphamide	127 (51.4)	98 (49.7)	29 (58.0)	0.297
Rituximab	53 (21.5)	43 (21.8)	10 (20.0)	0.779
Mycophenolate mofetil	68 (27.5)	55 (27.9)	13 (26.0)	0.786
Azathioprine	126 (51.0)	104 (52.8)	22 (44.0)	0.267
Tacrolimus	21 (8.5)	12 (6.1)	9 (18.0)	0.007
Methotrexate	31 (12.6)	30 (15.2)	1 (2.0)	0.008

Values are expressed as a median (25–75 percentile) or *N* (%). MPA: microscopic polyangiitis; GPA: granulomatosis with polyangiitis; BMI: body mass index; AAV: ANCA-associated vasculitis; ANCA: antineutrophil cytoplasmic antibody; MPO: myeloperoxidase; P: perinuclear; PR3: proteinase 3; C: cytoplasmic; BVAS: the Birmingham vasculitis activity score; FFS: the five-factor score; ESR: erythrocyte sedimentation rate; CRP: C-reactive protein; CALLY: C-reactive protein-albumin-lymphocyte; ESKD: end-stage kidney disease.

**Table 2:** Univariable and multivariable Cox proportional hazard analyses of variables at diagnosis for future progression to ESKD during the follow-up period in patients with MPA and GPA.

Variables	Univariable			Multivariable (CALLY Index)		
	HR	95% CI	*p* Value	HR	95% CI	*p* Value
**Demographic data**						
Age (years)	1.022	0.999, 1.045	0.057			
Male sex (*N*, (%))	1.280	0.687, 2.385	0.438			
BMI (kg/m^2^)	0.863	0.784, 0.951	0.003			
Ex-smoker (*N*, (%))	0.901	0.124, 6.539	0.918			
**ANCA type and positivity (** ***N*** **, (%))**						
MPO-ANCA (or P-ANCA)	2.462	1.100, 5.512	0.028	1.974	0.877, 4.447	0.101
PR3-ANCA (or C-ANCA)	0.617	0.263, 1.450	0.268			
**AAV-specific indices**						
BVAS	1.086	1.045, 1.129	<0.001	1.069	1.028, 1.111	<0.001
FFS	1.975	1.480, 2.636	<0.001			
**Laboratory results**						
White blood cell count (/mm^3^)	1.000	1.000, 1.000	0.228			
Haemoglobin (g/dL)	0.653	0.564, 0.756	<0.001			
Platelet count (×1000/mm^3^)	0.998	0.995, 1.000	0.089			
Blood urea nitrogen (mg/dL)	1.029	1.023, 1.035	<0.001			
Serum creatinine (mg/dL)	1.523	1.407, 1.648	<0.001	1.541	1.410, 1.683	<0.001
Serum total protein (g/dL)	1.012	0.959, 1.068	0.656			
Serum albumin (g/dL)	0.518	0.361, 0.744	<0.001			
**Acute phase reactants**						
ESR (mm/hr)	1.003	0.996, 1.010	0.365			
CRP (mg/L)	1.006	1.001, 1.010	0.013			
**Comorbidities (** ***N*** **, (%))**						
Hypertension	2.274	1.284, 4.028	0.005			
Type 2 diabetes mellitus	0.955	0.508, 1.797	0.886			
Dyslipidaemia	1.208	0.604, 2.417	0.592			
**CALLY index**	0.902	0.816, 0.997	0.043	0.997	0.943, 1.055	0.929

ESKD: end-stage kidney disease; MPA: microscopic polyangiitis; GPA: granulomatosis with polyangiitis; BMI: body mass index; MPO: myeloperoxidase; P: perinuclear; PR3: proteinase 3; C: cytoplasmic; AAV: ANCA-associated vasculitis; ANCA: antineutrophil cytoplasmic antibody; BVAS: the Birmingham vasculitis activity score; FFS: the five-factor score; ESR: erythrocyte sedimentation rate; CRP: C-reactive protein; CALLY: C-reactive protein-albumin-lymphocyte.

## Data Availability

The original contributions presented in this study are included in the article material. Further inquiries can be directed to the corresponding author.

## Supplementary Materials

The following supporting information can be downloaded at: https://www.mdpi.com/article/10.3390/medicina62071379/s1, Table S1: Comparison of the median earliest CALLY index calculated at diagnosis according to the presence of each systemic item of BVAS in patients with MPA and GPA. Figure S1: Comparison of the cumulative CVA- and ACS-free survival rates.

## Abbreviations

The following abbreviations are used in this manuscript:

AAV	antineutrophil cytoplasmic antibody-associated vasculitis
ACR	the American College of Rheumatology
ANCA	antineutrophil cytoplasmic antibody
AUC	area under the curve
BMI	body mass index
BVAS	the Birmingham Vasculitis Activity Score
C	cytoplasmic
CALLY	the C-reactive protein-albumin-lymphocyte
CRP	C-reactive protein
EGPA	eosinophilic granulomatosis with polyangiitis
ESKD	end-stage kidney disease
ESR	erythrocyte sedimentation rate
EULAR	the European Alliance of Associations for Rheumatology
FFS	the five-factor score
GPA	granulomatosis with polyangiitis
IRB	the Institutional Review Board
MPA	microscopic polyangiitis
MPO	myeloperoxidase
P	perinuclear
PR3	proteinase 3
ROC	receiver operating characteristic
RR	relative risk
