# Supplementary material for: Association Between the Earliest CALLY Index at Diagnosis and Progression to End-Stage Kidney Disease in Patients with Microscopic Polyangiitis and Granulomatosis with Polyangiitis
Source: Medicina (Kaunas). 2026 Jul 17;62(7):1379. doi: 10.3390/medicina62071379 (PMC13414433; doi:10.3390/medicina62071379)
Supplement: Supplementary file 1 [file medicina-62-01379-s001.zip › medicina-4352409-supplementary.pdf]

Table S1. Comparison of the median earliest CALLY index calculated at diagnosis according to the presence of each systemic item of BVAS in patients with MPA and GPA.

Variables	N, (%)	Absence	Presence	P-value
General manifestation	105 (42.5)	0.98	0.14	<0.001
Cutaneous manifestation	36 (14.6)	0.40	0.47	0.430
Mucous/Ocular manifestation	17 (6.9)	0.41	0.94	0.283
Ear nose throat manifestation	89 (36.0)	0.43	0.40	0.444
Pulmonary manifestation	154 (62.3)	0.47	0.30	0.024
Cardiovascular manifestation	46 (18.6)	0.53	0.16	0.020
Gastrointestinal manifestation	7 (2.8)	0.43	0.24	0.968
Renal manifestation	166 (67.2)	1.83	0.24	<0.001
Neurological manifestation	59 (23.9)	0.46	0.28	0.939

CALLY: C-reactive protein-albumin-lymphocyte; BVAS: the Birmingham vasculitis activity score; MPA: microscopic polyangiitis; GPA: granulomatosis with polyangiitis.

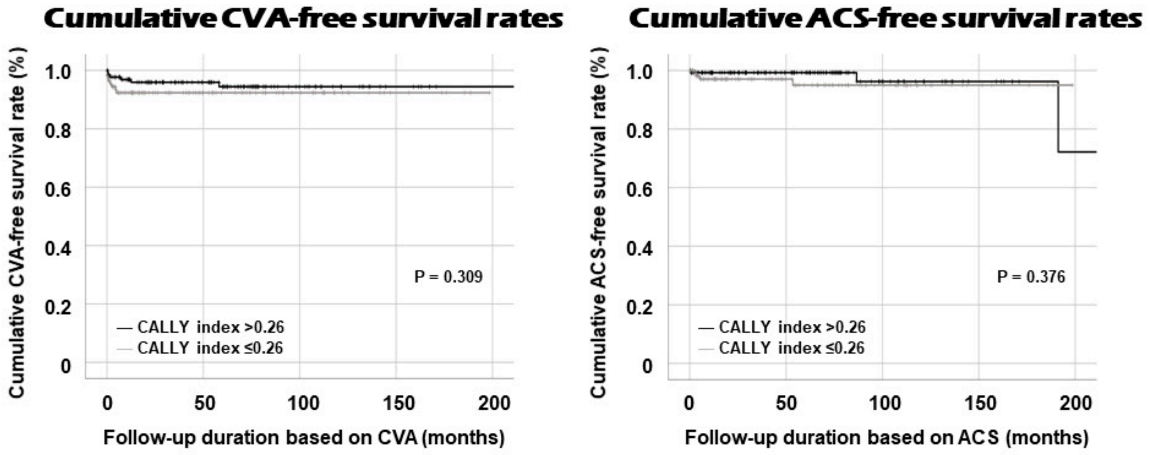

Figure S1. Comparison of the cumulative CVA- and ACS-free survival rates.
